# Supplementary figures and images for: Using search trends to analyze web-based users’ behavior profiles connected with COVID-19 in mainland China: infodemiology study based on hot words and Baidu Index
Source: PeerJ. 2022 Nov 9;10:e14343. doi: 10.7717/peerj.14343 (PMC9653070; doi:10.7717/peerj.14343)

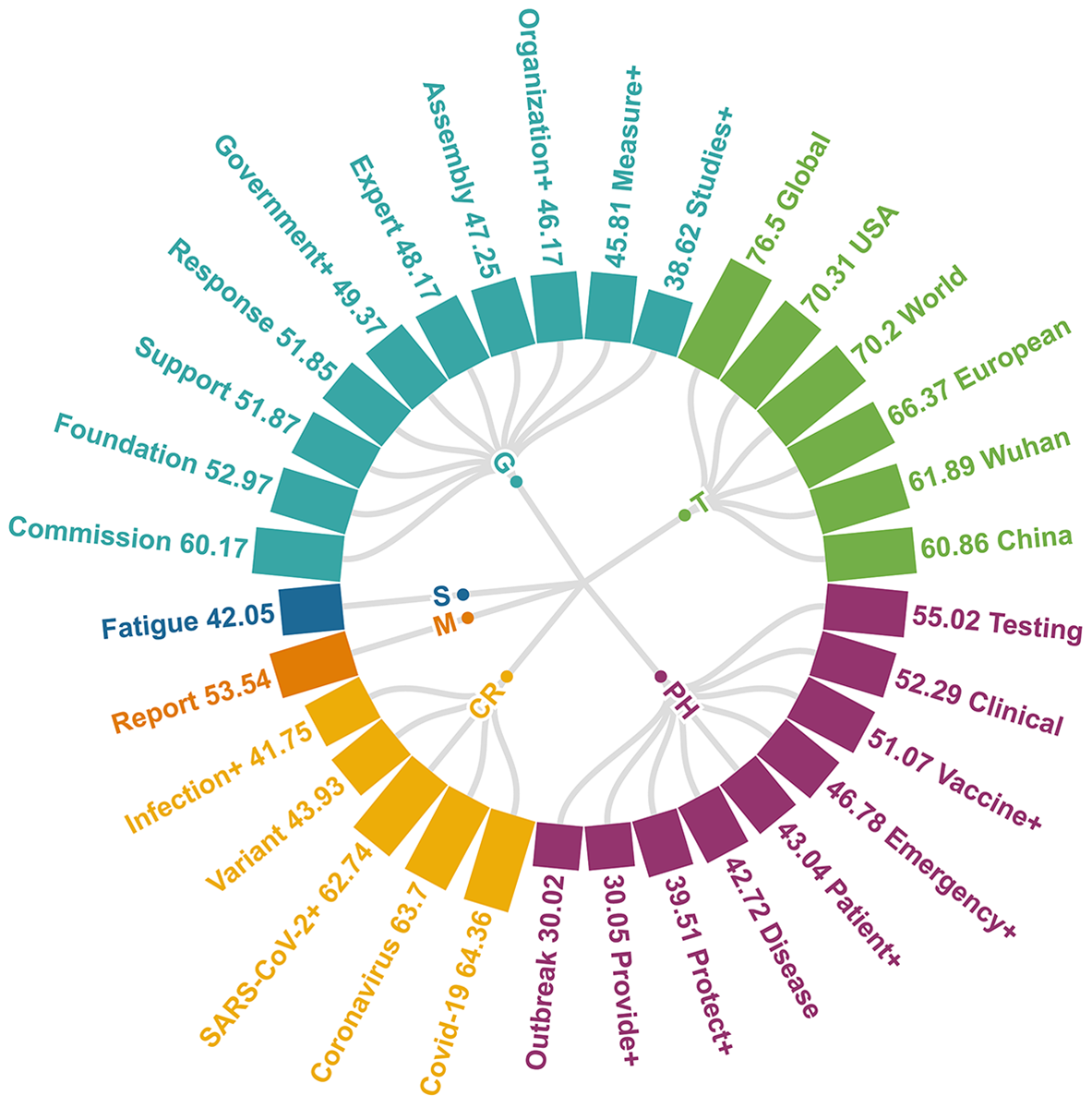

Supplement: Figure S1 [file peerj-10-14343-s001.png]

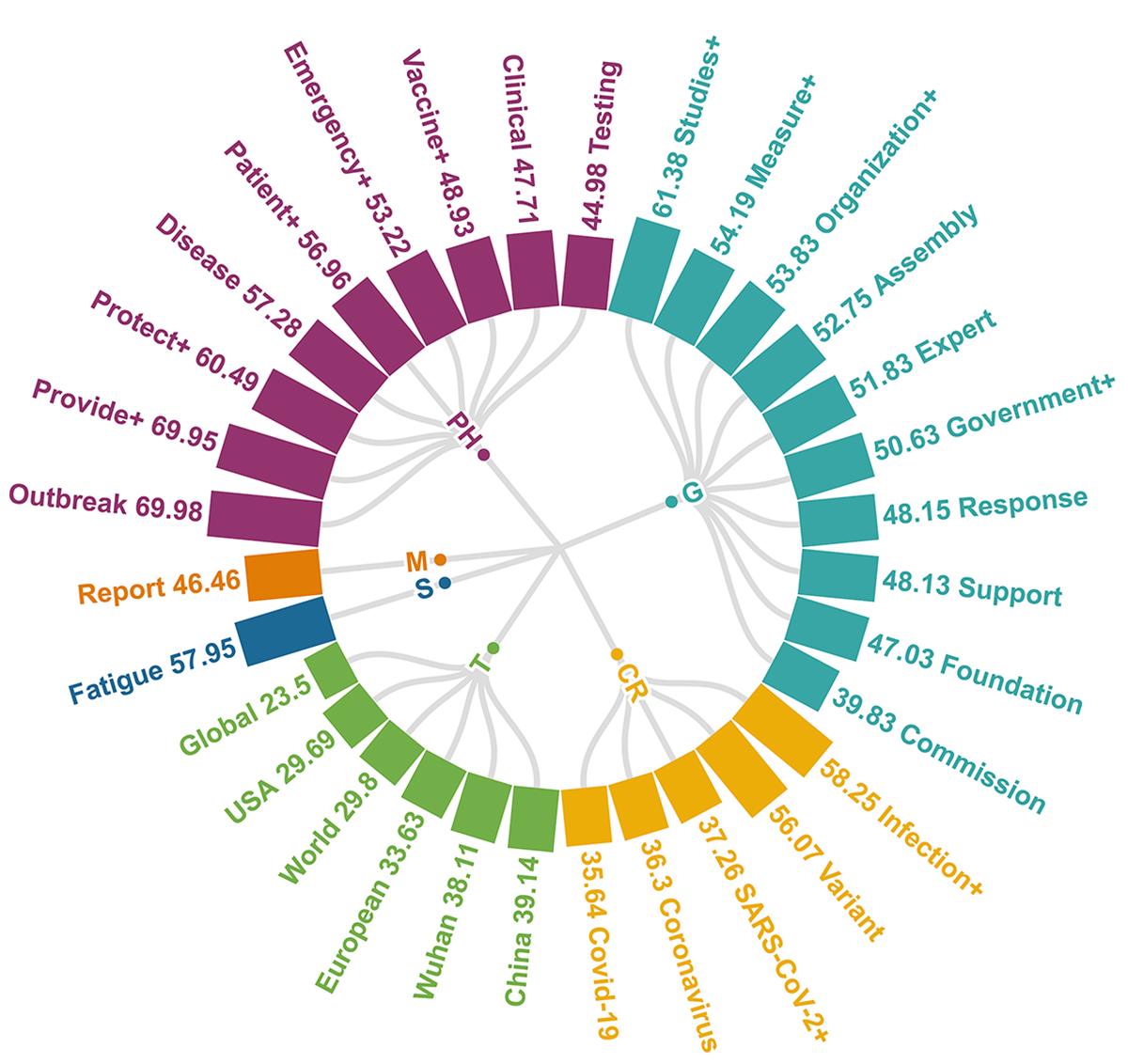

Supplement: Figure S2 [file peerj-10-14343-s002.png]

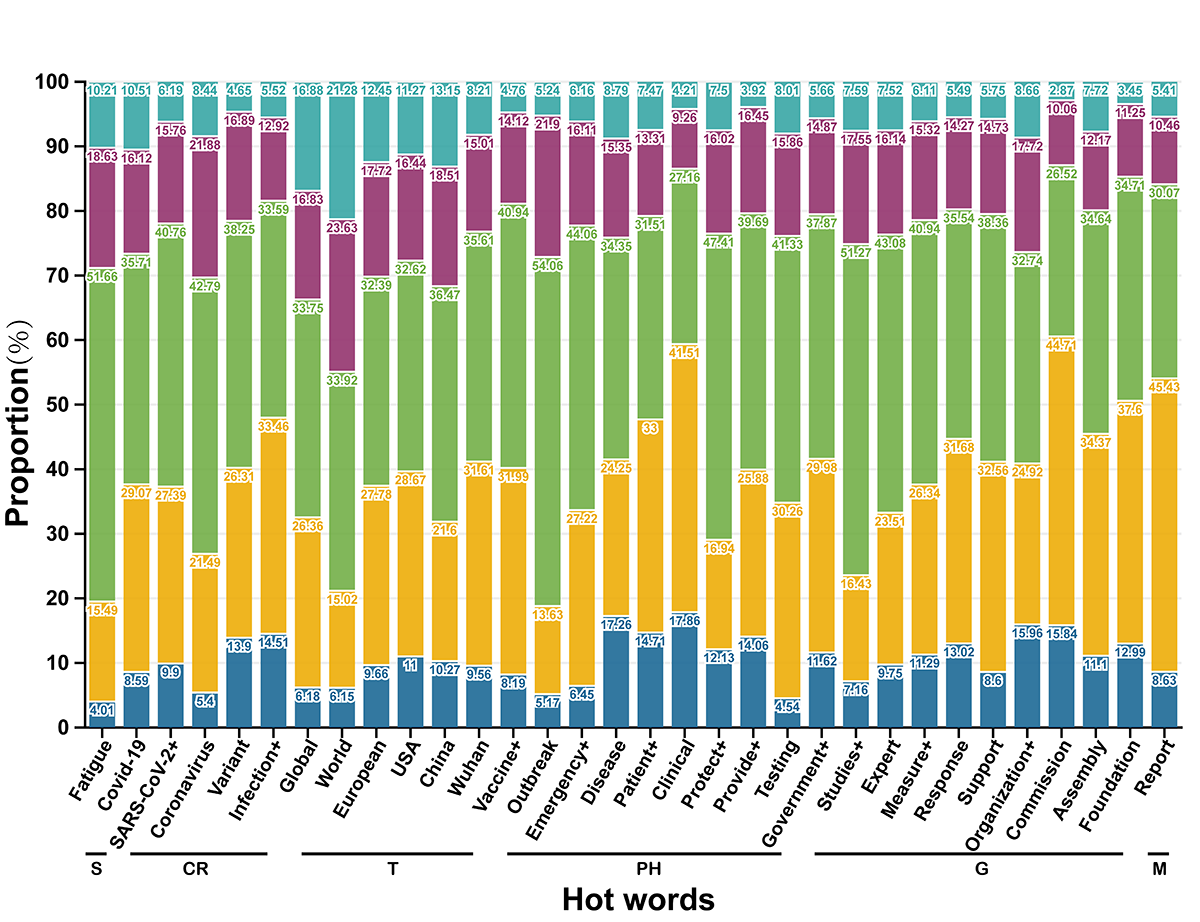

Supplement: Figure S3 [file peerj-10-14343-s003.png]
